# Clinical and Epidemiological Aspects of Multiple Sclerosis in Children

**Published:** 2017

**Authors:** Mohammad Mehdi NASEHI, Mohammad Ali SAHRAIAN, Abdorreza NASER MOGHADASI, Mohammad GHOFRANI, Fereshteh ASHTARI, Mohammad Mahdi TAGHDIRI, Seyed Hassan TONEKABONI, Parvaneh KARIMZADEH, Mahdi AFSHARI, Mahmood MOOSAZADEH

**Affiliations:** 1 Pediatric Neurology Research Center, Shahid Beheshti University of Medical Sciences, Tehran, Iran.; 2 Pediatric Neurology Excellence Center, Pediatric Neurology Department, Mofi Children Hospital, Faculty of Medicine, Shahid Beheshti University of Medical Sciences, Tehran, Iran; 3 Ms Research Center, Neuroscience Institute, Tehran University of Medical Sciences, Tehran, Iran; 4 Department of Neurology, Isfahan University of Medical Sciences, Isfahan, Iran.; 5 Department of Community Medicine, Zabol University of Medical Sciences, Zabol, Iran.; 6 Health Sciences Research Center, Faculty of Health, Mazandaran University of Medical Sciences, Sari, Iran.

**Keywords:** Multiple sclerosis, Children, Epidemiology, Clinical, Iran

## Abstract

**Objective:**

Overall, 2%-5% of patients with multiple sclerosis (MS) experienced the first episode of disease before the age 18 years old. Since the age of onset among children is not similar to that in general population, clinicians often fail to early diagnose the disease. This study aimed to determine the epidemiological and clinical patterns of MS among Iranian children.

**Materials & Methods:**

In this cross-sectional study carried out in Iran in 2014-2015, information was collected using a checklist with approved reliability and validity. Method sampling was consensus. Data were analyzed using frequency, mean and standard deviation indices by means of SPSS ver. 20 software.

**Results:**

Totally, 177 MS children were investigated. 75.7% of them were female. Mean (SD), minimum and maximum age of subjects were 15.9 (2), 7 and 18 yr, respectively. The most reported symptoms were sensory (28.2%), motor (29.4%), diplopia (20.3%) and visual (32.8%). Primary MRI results showed 91.5% and 53.1% periventricular and spinal cord lesions, respectively.

**Conclusion:**

MS is significantly more common among women. The most common age of onset is during the second decades. Visual and motor problems are the most symptoms, while, periventricular and spinal cord lesions are the most MRI results.

## Introduction

Multiple Sclerosis (MS) is one of the most common morbidities among young adults identifies with central nervous system demyelination (1, 2). It was described pathologically for the first time in the 19th century (3). MS is associated with inflammation and degeneration of the myelin in CNS in the form of small and large single or multiple plaques and can lead to different complications such as reduced visual acuity, spastic paralysis and disability, sphincter disorders, impotence especially among men, imbalance, speech disorder, seizure, action tremor and depression (4, 5).

The main cause of MS is unknown and different etiologic factors have been reported.

Various genetic, racial, environmental, infectious, toxic, immunologic and allergic factors were revealed (6-9). Patients complain of a wide spectrum of signs and symptoms from mild to moderate and transient to persistent. Many of them show brain stem involvement such as vertigo, diplopia, cranial nerve paralysis, eye movement disorder, sensory, and motor symptoms as the first manifestations. It begins as local or sectional myelin degeneration in the central nervous system (CNS) among youth follows by irregular periods of cure and remission (2, 3, 5).

Periodic neurologic attacks, disability and reduced physical, socio-economic and health quality during a 30-40 yr period of life is the main characteristics of MS (5). According to the disease trend and symptoms, it is characterized by relapsing-remitting type (70%), primary progressive and secondary progressive types (2, 10, 11).

Prevalence of MS among women is four times of that among men (2). It normally occurs among young adults. 

Approximately, two-thirds of cases begin between ages 20-30 yr, most probably in 30-year-old age. However, it has been reported even among two-year-old children (2, 3, 8, 12).

According to a longitudinal study conducted in a Colombian hospital, 116 MS cases were identified before the age of 16 yr. Mean age of onset was 12.73 yr and the prevalence of early MS was 3.6% (13). 

Maximum and minimum ages of onset were 13 and 73 yr, respectively in Isfahan (14) and 4 and 13 yr, in Tehran (3).

Since the age of onset among children is not in accordance with the common age of the disease, clinicians often fail to diagnose the disorder in the early stages. In addition, differential diagnosis of MS among children is of great importance and may be ignored during childhood leading to mental, psychological and economical problems for the families. Few relevant studies have been carried out. Different studies (2-3, 14-16) reported heterogenic results making it difficult to implement appropriate strategies. This study aimed to investigate comprehensively the epidemiological and clinical pattern of MS among Iranian children. 

## Materials & Methods

This cross-sectional study was conducted in 2015 among Iranian children suffering from MS selected by consensus method. After approving by Ethics Committee of Shahid Beheshti University of Medical Sciences, primary information of patients aged under 18 yr were collected from relevant clinical centers in Tehran (Sian Hospital, Mofid Hospital, Clinic of Dr. Sahraian, Clinic of Dr. Moghaddasi), Esfahan (Clinic of Dr Ashtari).

Using a checklist including demographic and clinical variables such as age, gender, residence area, familial history, clinical manifestations at the onset and relapse, number of relapses, the time interval between the first attack and relapse and MRI results (initial and after three months). This checklist was designed based on previous similar studies (2-4, 14-16). Content validity was assessed according to the expert panels. To evaluate the reliability, objective items of the checklist (clinical characteristics) were completed for 10 patients and repeated after 10 d. The correlation between the answers was determined as of 0.79 using Spearman correlation. 

Descriptive statistics such as frequency, mean, standard deviation (SD) and median were estimated using SPSS software (Chicago, IL, USA). 

## Results

We recruited 177 MS children most of them were girl (75.7%). Mean±SD, minimum and maximum ages of them were 15.9±2, 7 and 18 yr, respectively. Of them, 35.6% were living in Tehran, 8.5% in Isfahan, 8.5% in Mazandaran and 32.7% in the other provinces. The residence area of the 26 (14.7%) were unknown. Moreover, 16.4% had familial history of MS (Table 1). The most common manifestations at the onset were impaired or loss of vision (32.8%), motor (29.4%) and sensory (28.2%) disorders. During the relapse, 31.6%, 30.5%, and 22.6% had sensory problems, motor problems and loss of vision, respectively (Table 2).

Among them, 17.5% reported more than three attacks. The time interval between the first attack and relapse was more than one year for 52.2% of patients (Table 3). Table 4 illustrates the MRI results.

**Table 1:** Demographic Characteristics of the Studied Patients by Gender, Residence Area and Family History

**Variables**		**Number**	**Percent**
**Gender**	**Male**	43	24.3
	**Female**	134	75.7
**Residence area**	**Tehran**	61	35.6
	**Isfahan**	15	8.5
	**Mazandaran**	15	8.5
	**Other provinces**	60	32.7
	**Unknown**	26	14.7
**Family history**	**First-degree relatives**	8	4.5
	**Second-degree relatives**	21	11.9
	**Total family history**	29	16.4
**Total**		177	100

**Table 2:** Clinical Characteristics of the Study Population by Symptoms

**Symptoms **	**At the onset**		**During the relapse**	
	**Number (of 177)**	**Percent**	**Number (of 177)**	**Percent**
**Diplopia**	36	20.3	18	10.2
**Loss of vision**	58	32.8	40	22.6
**Color vision disorder**	0	-	2	1.1
**Scotoma**	1	0.6	0	-
**Eye movement disorder**	1	0.6	3	1.7
**Ataxia**	28	15.8	40	22.6
**Dysarthria**	3	1.7	5	2.8
**Cranial nerve palsy**	7	4	7	4
**Sensory **	50	28.2	55	31.6
**Motor**	52	29.4	54	30.5
**Behavioral disorder**	1	0.6	2	1.1
**Seizure**	5	2.8	6	3.4
**Defecation and urinary problems**	2	1.1	10	5.6

**Table 3:** Number of Attacks and Time between Onset and Relapse

**Variables**		**Number**	**Percent**
**Number of attacks**	**Single attack**	4	2.3
	**2-3**	34	19.2
	**More than three**	31	17.5
	**No attack**	108	61
**Total**		177	100
**Time between onset and relapse**	**Less than 6 months**	21	30.4
	**6-12 months**	5	7.2
	**More than 12 months**	36	52.2
	**Unknown**	7	10.1
**Total**		69	100

**Table 4:** MRI Results of the Study Population

**Results** **MRI**		**At the onset**		**After 3 months**	
		**Number**	**Percent**	**Number**	**Percent**
**Number of lesions**	**<2**	12	6.8	42	31.5
	**>=2**	139	78.5	91	68.4
	**total**	151	100	133	100
**Periventricular lesion**	**yes**	161	91.5	107	62.2
	**no**	15	8.5	65	37.8
	**total**	176	100	172	100
**Juxtacortical lesion**	**yes**	39	22	21	12.2
	**no**	138	78	151	87.8
	**total**	177	100	172	100
**Infrateutorial lesion**	**yes**	20	11.3	22	12.8
	**no**	157	88.7	150	87.2
	**total**	177	100	172	100
**Spinal cord involvement**	**yes**	94	53.1	67	39.2
	**no**	83	46.9	104	60.8
	**total**	177	100	171	100
**Number of spinal cord lesions**	**<=2**	28	34.1	16	26.7
	**>2**	54	65.9	44	73.3
	**total**	82	100	60	100
** Presence of blackhole**	**yes**	1	0.6	1	0.6
	**no**	176	99.4	171	99.4
	**total**	177	100	172	100
**Enhancing lesion**	**yes**	29	16.4	23	13.5
	**no**	148	83.6	148	86.6
	**total**	177	100	171	100

## Discussion

Iranian women developed MS more than three folds greater than Iranian men did and mean age of the patients was approximately 16 yr. The most common signs and was symptoms during initial and relapse phases of disease were visual problems, ataxia and sensory-motor dysfunction. More than three attacks were observed among 17.5% of patients. More than half of them experienced some evidence of relapse at least one year after the first attack. Familial history of MS was reported by 16.4% of them. MRI results revealed that most of patients (78.5%) had at least two lesions and 37.5% had three or more plaques. While periventricular lesions (91.5%) and spinal cord lesions (53.1%) were observed among MS patients.

From the viewpoint of gender distribution, the prevalence of MS among women in northern part of Iran was 2-4 folds greater than that among men (2). Corresponding figures for women and men were reported as of 70 and 30 per 100000 population (3), 77.4 and 22.6 per 100000 population in Hamadan, Iran (15), 65.6 and 21.9 per 100000 population in Kermanshah, Iran (16) MS typically occurs among young and middle age groups. In two-thirds of cases, it starts during ages 20 to 40 yr. However, even two-year-old children have experienced this disorder (2, 3, 8, 12). Clinical presentations are almost similar for children and adults. 

MS remains harmful complications on different aspects of children lives. In addition, MRI diagnostic criteria in children are similar to those of adults (3, 8, 12). During a longitudinal study carried out in a Colombian hospital, 116 incident cases were reported. The age of onset was before 16 for all cases with mean age of 12.73 yr 3.6% of them were early onset (17). Prevalence of early MS was reported as 19.8% in Jordan (10) and 3.6% in Russia (10). A retrospective study among 20 Iranian cases reported four and 13 yr as minimum and maximum age of onset (3). Since the age of onset among children is not in parallel with that among adults, clinicians often fail to early diagnose the pediatric MS. Two to five percent of cases experienced the first symptoms before 16 yr (18). The incidence of early MS before 16 has been reported as 0.4% to 10.5% globally (17). It can be an explanation for the observed disagreement between the age patterns and our results.

Most of the above studies carried out among patients from Capital, Northern and central provinces of Iran.

Prevalence of MS was reported as 20.1 (95% confidence interval: 18.7-22.1) per 100000 population, among 582 patients. Therefore, Mazandaran- Northern Province of Iran- is one of the regions with moderate to high prevalence (2). Isfahan (19) and Kermanshah (15) in Iran were reported as the high prevalence provinces with 35.5 and 43.3 cases per 100000 population, respectively (8, 10). The prevalence of MS was reported as 73 per 100000 population in the world and 60 per 100000 population in Iran (16). The prevalence of MS in Isfahan was reported as 43.5 per 100000 population (20). Although the above reports are in keeping with the results of the current study, it requires more investigations, because prevalence was not reported from all parts of the country.

Among 20 cases (14 girls and 6 boys) in Mofid Hospital, all patients showed increased patellar reflexes. In twothirds of them (13 cases), muscle strength had been decreased. Impaired cerebellar tests, diplopia, and ataxia were observed in half (10 cases), two-thirds (14 cases) and two-thirds (13 cases) of MS patients. MRI results were abnormal in all cases. Spinal MRI was performed for seven patients five of which showed hyper signal results in spinal canal. All patients had normal CSF (3).

The epidemiological and clinical characteristics of early and late onset MS cases were investigated (155 patients including 2.6% men and 77.4% females) living in Hamadan Province (western province of Iran). The first manifestations were optic neuritis in 54 cases (34.84%), motor disorders in 45 cases (29.04%), sensory manifestations in 38 cases (24.51%) and balance disorders (cerebellar and brainstem involvement) in 25 cases (14.83%). Moreover, 25 patients (16.10%) showed simultaneously more than two manifestations at the onset of disease. Among early onset patients, 24 cases (14.83%) aged 18 or less, but no clinical or epidemiological differences were observed between children and adults. In addition, 12.9% of patients had familial history of MS (15).

In Kermanshah (western Iran), among 448 MS cases, only five (1.2%) of patients had familial history of disease among their first-degree relatives including father (one case), sister (three cases) and son (one case).

History of MS among second-degree relatives was reported for nine (2.1%) of cases. Definitive diagnosis had been done after six months or earlier in 42%, after 6-11 months in 9.2%, after 1-2 yr in 19.4%, after 3-4 yr in 8% and after five yr in 7.1% of cases. The most common signs and symptoms at the onset of disease were sensory (25.2%) and visual (19.4%) problems (16).

Of 203 patients who were investigated, the first manifestations were sensory-motor disorders in 58(28.6%) cases, visual problems in 50 (24.4%), visual-sensory-motor involvement in 48 (23.6%), motor disorder in 19(9.4%), cerebellar disorders in 11(5.4%), sensory and visual problems in 8(3.9%), urination and defecation problems in 4(2%) and brain stem involvement in 2 (1%) cases (21). MS patients were investigated in Isfahan (center of Iran), 20.1% had positive familial history of MS, 40.5% of which were in the first-degree relatives (14). The above-mentioned results are in occurance with those found in our study.

There were some limitations in the current study. We did not have access to the patients’ information of all provinces in Iran, therefore, a geographical pattern was not provided by this study. The observed age of disease onset was not reliable but all cases have developed the disease before age 18. Finally, defects and high variations in patients’ records made the research process slightly difficult.

**In conclusion, **MS is considerably more common among women and starts commonly from the second decade of life. Moreover, sensory-motor manifestations, loss of muscle strength and level of consciousness were dominant signs and symptoms of the disease, while, periventricular and spinal cord lesions are the main MRI results during the MS development. To implement effective treatment strategies and strengthen the research foundations, it is necessary to design a comprehensive MS surveillance system within the community.
